# Two different right ventricular pacing waveforms

**DOI:** 10.1093/ehjcr/ytae119

**Published:** 2024-03-04

**Authors:** Toshinori Komatsu, Ayako Okada, Koichiro Kuwahara

**Affiliations:** Department of Cardiovascular Medicine, Shinshu University School of Medicine, Asahi 3-1-1, Nagano 390-8621, Japan; Department of Cardiovascular Medicine, Shinshu University School of Medicine, Asahi 3-1-1, Nagano 390-8621, Japan; Department of Cardiovascular Medicine, Shinshu University School of Medicine, Asahi 3-1-1, Nagano 390-8621, Japan

## Clinical vignette

A 75-year-old woman presented to our hospital with a 1-year history of shortness of breath and bipedal oedema. She had a dual-chamber pacemaker implanted 2 years previously because of a complete atrioventricular block; she was asymptomatic at the time of implantation. At presentation, the patient’s vital signs were within normal limits, but physical examination revealed significant bipedal oedema. She was severely obese, with a body mass index (BMI) of 39 kg/m^2^. Pulse irregularities, rales, or heart murmurs were absent. Transthoracic echocardiography revealed normal left ventricular systolic function. The pacemaker was programmed in the DDD mode at 60–130 beats/min and atrial-paced atrioventricular delay at 180 ms. A 12-lead electrocardiogram (ECG) was obtained and is shown below.

**Figure:**
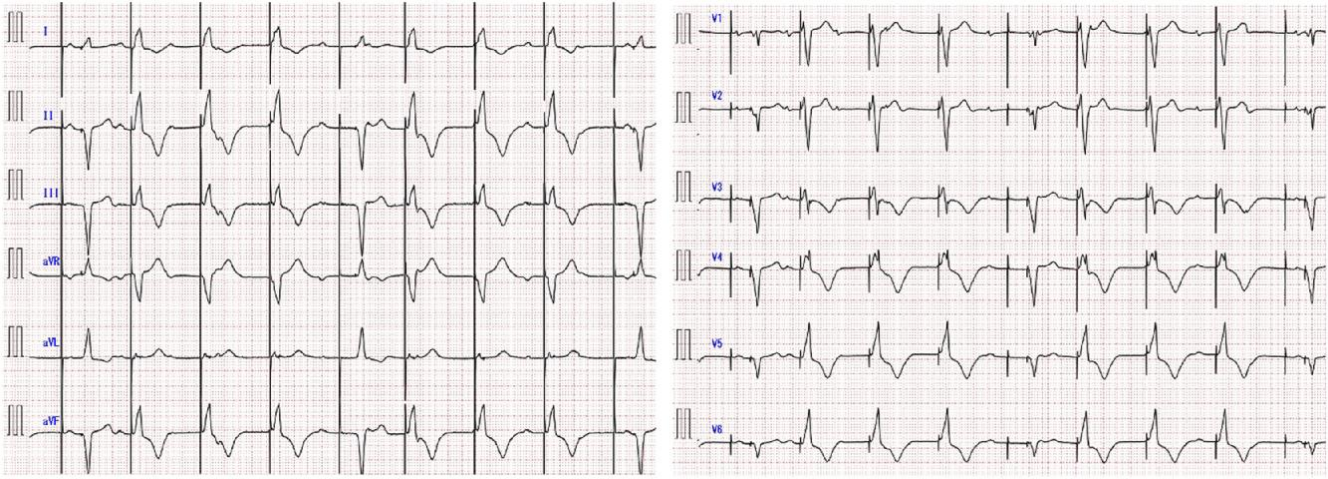


## Question 1

What is the most likely diagnosis in this ECG?

- Normal right atrial (RA) sense
- Right ventricular (RV) pacing failure
- RV pacing and frequent premature ventricular contraction (PVC)
- Dual-site RV pacing
- Sinus rhythm (SR) and RV pacing

The correct answer is D.

## Explanation

Symbols were added to the pacing spikes on the ECG on admission (see Supplementary material online, *Figure S1*). Preceding RA pacing spikes were not observed, and atrial activity originated from the sinus node. However, the PR interval was not constant. Therefore, SR was not observed, and RA sensing failure occurred; thus, A and E are incorrect. Two types of R-waves were identified, each following two different pacing spikes, indicating no RV pacing failure. Therefore, B is incorrect, and D is correct. All R-waves were not PVCs because a preceding pacing spike was observed. Therefore, C is incorrect.

## Question 2

Chest radiographs are shown in Supplementary material online, *Figure S2*. What is the best combination of lead positions to explain the ECG?

- RV apex and His-bundle pacing
- RV apex and left-bundle branch (LBB) pacing
- RV apex and RV outflow tract (RVOT) pacing
- RV apex and coronary sinus (CS) pacing
- Dual RV apex pacing

The correct answer is C.

## Explanation

One type was negative, and another was positive R-wave in leads II, III, and aVF. Chest radiograph findings suggested that the leads were at RV apex and RVOT. His-bundle, LBB, or CS pacing did not result in a LBB block. Thus, A, B, D, and E are incorrect, and C is correct. The pacemaker was programmed to VVI, the first pacing spike disappeared, and the second pacing spike was followed by a monomorphic R-wave, suggesting that it originated from the RV lead (see Supplementary material online, *Figure S3*). The first pacing spike was due to a dislodged RA lead that strayed into the RVOT.

## Question 3

Which is not a risk factor of lead dislodgement?

- Passive fixation leads
- Active fixation leads
- Female sex
- High BMI
- Low atrial septal lead position

The correct answer is B.

## Explanation

The risk factors for lead dislodgement are female sex and high BMI.^1^ Using passive fixation leads for RA leads increases the risk of lead dislodgement compared with using active fixation leads. The rate of lead dislodgement is higher in the RA septal position than in the free wall or appendage.^2^ Therefore, A, C, D, and E are incorrect, and B is correct. In this case, the tined RA lead that strayed into the RVOT had an unstable fixation. As an abandoned lead may cause life-threatening arrhythmias, transvenous lead extraction was performed and a new lead was placed.^3^

## Supplementary Material

ytae119_Supplementary_Data

## Notes

**Consent:** Direct written consent was obtained by the patient for this manuscript.

**Funding**: This report did not receive any specific grants from funding agencies in the public, commercial, or not-for-profit sectors.

**Data availability**: The data that support the findings of this study are available in the article and in its online supplementary material.

## References

[1] Qin D, Filippaios A, Murphy J, Berg M, Lampert R, Schloss EJ, et al Short- and long-term risk of lead dislodgement events: real-world experience from product surveillance registry. Circ Arrhythm Electrophysiol 2022;15:e011029. 35925831 10.1161/CIRCEP.122.011029

[2] Witt CM, Lenz CJ, Shih HH, Ebrille E, Rosenbaum AN, Aung H, et al Right atrial lead fixation type and lead position are associated with significant variation in complications. J Interv Card Electrophysiol 2016;47:313–319. 27613185 10.1007/s10840-016-0181-y

[3] Kusumoto FM, Schoenfeld MH, Wilkoff BL, Berul CI, Birgersdotter-Green UM, Carrillo R, et al 2017 HRS expert consensus statement on cardiovascular implantable electronic device lead management and extraction. Heart Rhythm 2017;14:e503–e551. 28919379 10.1016/j.hrthm.2017.09.001

