# Supplementary figures and images for: Two different right ventricular pacing waveforms
Source: Eur Heart J Case Rep. 2024 Mar 4;8(3):ytae119. doi: 10.1093/ehjcr/ytae119 (PMC10939170; doi:10.1093/ehjcr/ytae119)

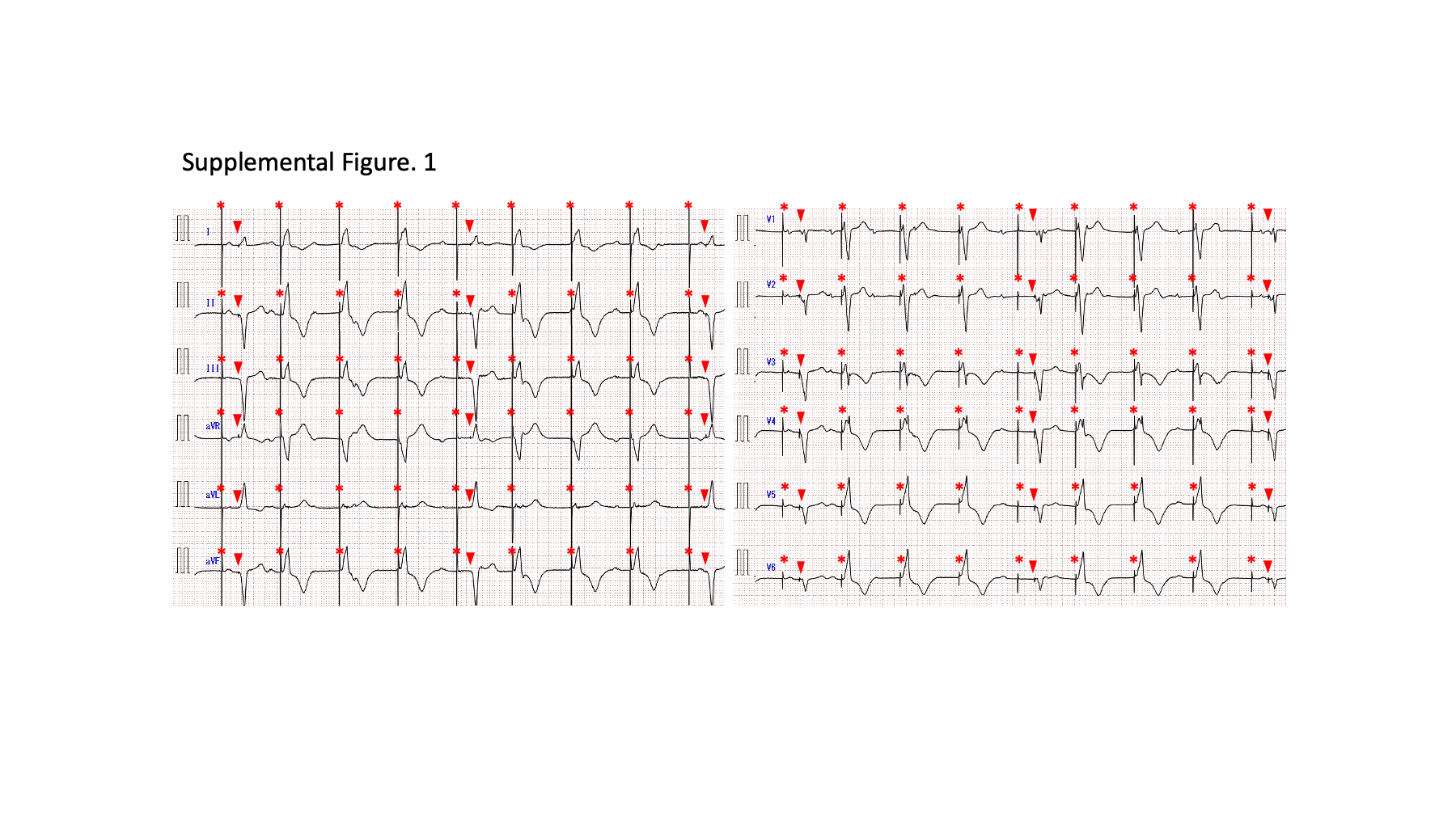

Supplement: ytae119_Supplementary_Data [file ytae119_supplementary_data.zip › S_Figure.1.tiff]

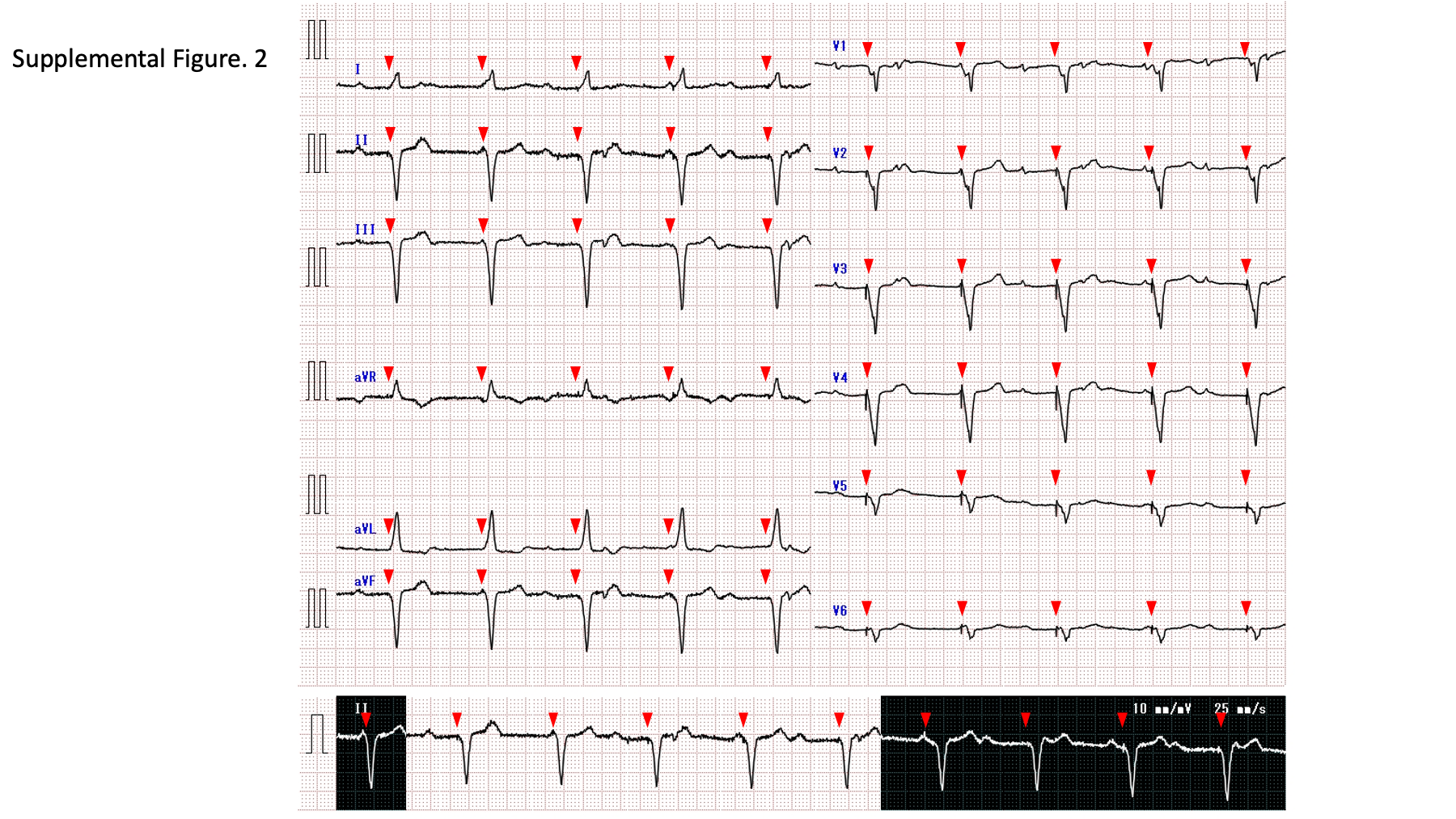

Supplement: ytae119_Supplementary_Data [file ytae119_supplementary_data.zip › S_Figure.2.tiff]

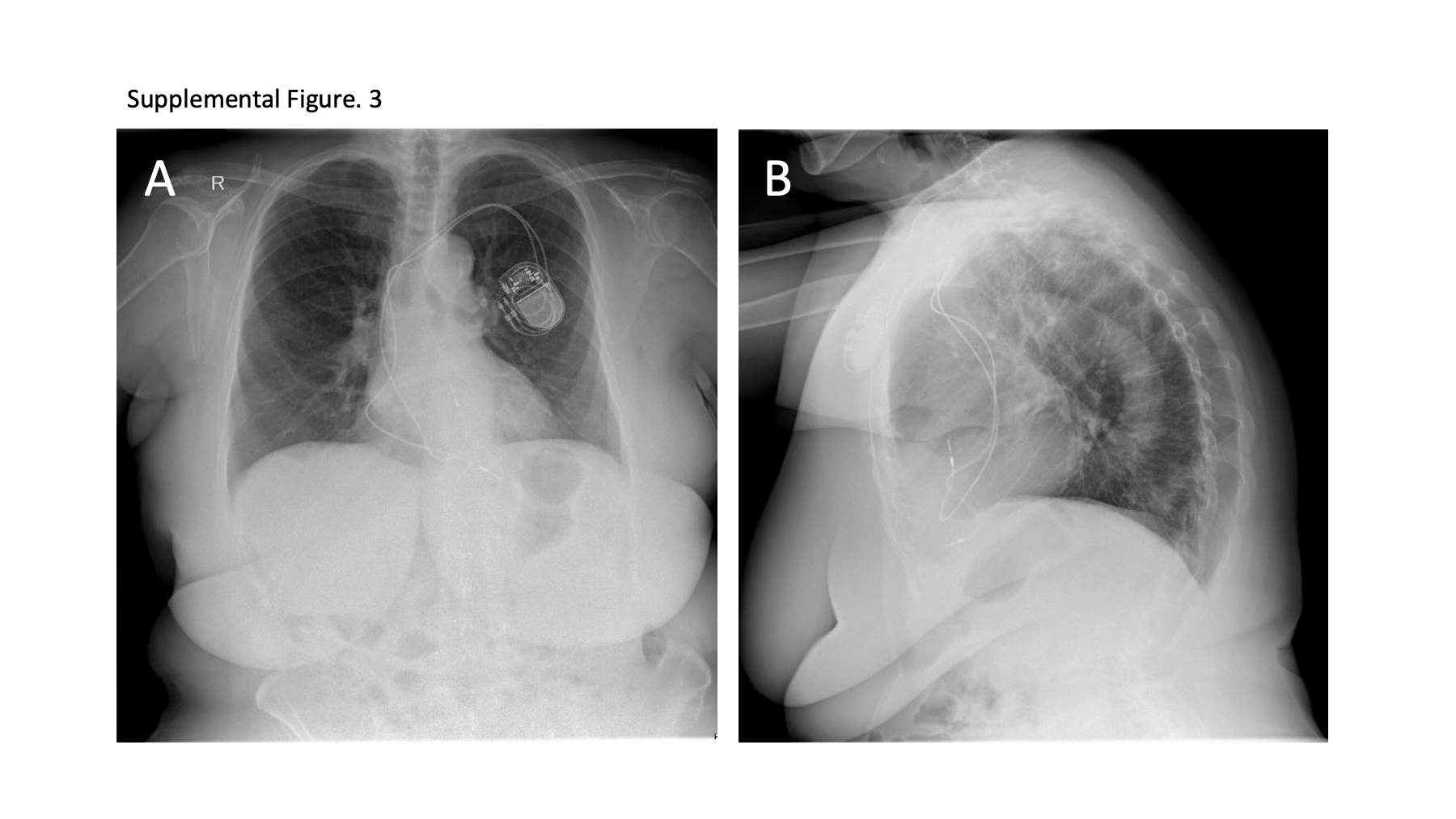

Supplement: ytae119_Supplementary_Data [file ytae119_supplementary_data.zip › S_Figure.3.tiff]
